# Histopathologic and Genomic Characterization of a Novel Caprine Astrovirus Identified in a Boer Goat Kid in Illinois, United States

**DOI:** 10.3390/v18010120

**Published:** 2026-01-16

**Authors:** Jingyi Li, Wes Baumgartner, Leyi Wang

**Affiliations:** Veterinary Diagnostic Laboratory, Department of Veterinary Clinical Medicine, College of Veterinary Medicine, University of Illinois, Urbana, IL 61802, USA

**Keywords:** goat, astrovirus, mNGS, histopathologic, genomic, characterization, identity

## Abstract

Astroviruses are non-enveloped, positive-sense single-stranded RNA viruses known to infect various mammals and birds, including humans, often causing gastrointestinal disorders. In recent years, astroviruses have also been linked to neurological and respiratory diseases across several species, including ruminants, mink, deer, and other mammals. Notably, astrovirus infections in goats have been documented in countries such as Switzerland and China, where novel genotypes have been identified in fecal samples. However, their role in the context of disease remains unclear, and reports focusing solely on goat astrovirus in the United States have not been published. A necropsy case of a Boer goat kid with a history of diarrhea was submitted for investigation following death in January 2025. Fresh tissues were received and used for histopathology and enteric pathogen testing, including parasitic, bacterial, and viral workups. Metagenomic-based next-generation sequencing (mNGS) was also applied for this case. Histological examination revealed severe necrotizing enterocolitis. The small intestine exhibited epithelial ulcerations, villus atrophy, hyperplastic and dilated crypts with necrotic debris, few intraenterocytic coccidian parasites, and increased inflammatory cells in the lamina propria. The large intestine showed similar findings with pleomorphic crypt enterocytes. Standard enteric pathogen tests were negative except for aerobic culture that identified *Escherichia.coli* and *Enterococcus hirae*. mNGS and bioinformatic analysis identified a novel astrovirus in the intestinal content that showed the highest nucleotide identity (86%) to the sheep strain Mamastrovirus 13 sheep/HA3 from China based on BLAST analysis. Phylogenetic analysis indicated that the newly identified caprine astrovirus IL90175 clustered with astrovirus strains from small ruminants in Asia and Europe. This research reports the discovery, histopathologic features, and genetic characteristics of a gastrointestinal disease-causing astrovirus in a goat kid, which had not been previously described in the United States.

## 1. Introduction

Astroviruses are small, non-enveloped, positive-sense single-stranded RNA viruses belonging to the family *Astroviridae* [1]. Within the family, the two genera *Mamastrovirus* and *Avastrovirus* encompass viruses infecting mammals and birds, respectively [2]. The genome of astroviruses is approximately 6–8 kb long, contains 5′ and 3′ untranslated regions, and three open reading frames (ORFs): ORF1a, ORF1b, and ORF2. The 5′ end is not capped, and ORF1a and ORF1b encode the astrovirus nonstructural proteins, including a serine protease, a viral genome-linked protein (VPg), and an RNA-dependent RNA polymerase (RdRp). At the 3′ poly (A) tail, ORF2 encodes the astrovirus capsid protein [3].

Human astrovirus in children with diarrhea was first described in 1975 [4]. Since then, more cases have been reported in a variety of species, including sheep, cattle, chickens, pigs, dogs, cats, red deer, ducks, mice, turkeys, mink, guinea fowl, bats, cheetahs, sea lions, and rats [1]. Astrovirus infection in different hosts is associated with gastroenteritis, nephritis, hepatitis, encephalitis, polioencephalomyelitis syndrome, and respiratory illness [1,5,6,7].

Transmission primarily occurs via the fecal–oral route. In most species, including humans and sheep, infections are typically asymptomatic or cause mild, self-limiting diarrhea, but in rare cases, infections may lead to severe diarrhea [8]. In turkeys, for example, astrovirus infection is associated with increased mortality and severe diarrhea, despite only mild histopathological lesions [9]. However, the causal relationship between astrovirus infection and diarrhea remains controversial in many species, in that astroviruses have been detected in both healthy and diarrheic animals, namely cattle, goats, roe deer, and pigs [10,11,12,13]. Nevertheless, a 2021 study demonstrated a strong correlation between astrovirus infection and diarrhea in cattle, both as a single agent and during co-infections [14]. In addition to gastrointestinal disease, astroviruses have also been implicated in neurological disorders in animals such as cattle and sheep, encephalomyelitis in pigs [2,15,16,17], as well as “shaking mink syndrome” [18]. Viral respiratory disease has been reported in white-tailed deer [6], pigs [19], and cattle [5].

Caprine astrovirus was identified initially in goat fecal samples from Switzerland in 2019, where three genotypes were discovered: caprine astrovirus G5.1, caprine astrovirus G3.1, and MAstV–34 [20]. In 2021, a novel genotype was detected in goats in China [21]. Subsequent surveillance in China in 2022 and 2024 identified three additional genotypes from goat feces (MAstV–13 and MAstV–24), both with unique genetic and epidemiological profiles [11,22]. These discoveries highlight the circulation of multiple, genetically diverse caprine astrovirus genotypes worldwide. To date, no research has characterized the genetics of goat astrovirus and their correlation with gastrointestinal disease in the United States. In this study, we report the first identification of a novel caprine astrovirus in the United States.

## 2. Materials and Methods

### 2.1. Necropsy Samples

In January 2025, necropsy tissues (lung, heart, spleen, kidney, small and large intestines, mesenteric lymph node, abomasum, skeletal muscle, rumen) from a 4-week-old female Boer goat kid were submitted to the University of Illinois Urbana–Champaign Veterinary Diagnostic Laboratory (UIUC-VDL) for histopathological examination and microbiological identification.

### 2.2. Histopathology Preparation

The necropsy tissues were fixed in 10% neutral buffered formalin, trimmed into cassettes, and routinely processed. All tissues were evaluated with routine hematoxylin and eosin (H&E) staining.

### 2.3. Routine Laboratory Testing

Both the gross lesions and histopathological findings suggested infectious causes, and intestinal tissues were submitted for pathogen testing, including bacterial culture, *Salmonella* PCR (DuPont Qualicon BAX^®^ System, Hygiena, Wilmington, DE, USA), rotavirus antigen assay (SAS™ Rota Test, SA Scientific, Ltd., San Antonio, TX, USA), and fecal flotation (Sheather’s solution) for parasites. Both *Salmonella* PCR and rotavirus antigen assay were performed following the manufacturer’s directions.

### 2.4. Intestinal Content Suspension Preparation and Nucleic Acid Extraction

Nucleic acids were extracted from intestinal content. The intestine lumen was swabbed with a cotton-tipped wooden applicator. The swab tip was then placed in a microcentrifuge tube containing 1000 µL PBS buffer, agitated, and removed. The tube was vortexed for 20 s and centrifuged for 2 min at 6000× *g* and room temperature. The nucleic acid extraction was performed using the MagMAX Pathogen RNA/DNA Kit (ThermoFisher, Waltham, MA, USA) on a KingFisher Flex machine (ThermoFisher, Waltham, MA, USA) [23].

### 2.5. Metagenomics-Based and Targeted-Based Next-Generation Sequencing (NGS), Assembly, and Analysis

#### 2.5.1. Metagenomics-Based NGS (mNGS)

The extracted nucleic acid was subjected to sequence-independent, single primer amplification (SISPA) as previously described [24]. It was first reverse transcribed into complementary DNA (cDNA) using Superscript III reverse transcriptase (ThermoFisher, Waltham, MA, USA) with a random octamer primer containing a defined sequence tag. Double-stranded DNA (dsDNA) was then synthesized using Klenow polymerase (NEB, Ipswich, MA, USA). Subsequent amplification was carried out with a single primer corresponding to the sequence tag using the Advantage 2 PCR kit (Takara Bio, Ann Arbor, MI, USA). The resulting PCR amplicons were purified with the QIAquick PCR Purification Kit (QIAGEN, Germantown, MD, USA) and quantified with the Qubit high-sensitivity assay kits (ThermoFisher, Waltham, MA, USA).

Library preparation was performed using the Nextera XT DNA Library Preparation Kit (Illumina, San Diego, CA, USA) according to the manufacturer’s instructions. The procedure included tagmentation, PCR amplification, PCR cleanup, library normalization, and final pooling. The sample was sequenced using an Illumina MiSeq v2 sequencing kit (300 cycles) on MiSeq (Illumina, San Diego, CA, USA).

#### 2.5.2. Targeted-Based NGS (tNGS)

The assembled genome had two contigs with a 61 bp gap within the capsid region. To close the gap and confirm the capsid sequence, three pairs of primers were designed and used for amplicon-based sequencing of the whole capsid region (Table 1). One-step RT-PCR was performed using the SuperScript™ III One-Step RT-PCR System with Platinum™ Taq DNA Polymerase (ThermoFisher, Waltham, MA, USA) and 5 µL RNA in a 25 µL reaction volume. Amplicon was purified using the QIAquick PCR Purification Kit (QIAGEN, Germantown, MD, USA) and sequenced on MiSeq as described in Section 2.5.1.

#### 2.5.3. Bioinformatic Sequence Analysis

Raw FASTQ files from MiSeq were initially analyzed using Kraken taxonomic classification software (Kraken 2 version) [25] using a database built through a standard method and then assembled using the de novo SPAdes assembler (v4.0.0) [26]. The resulting contigs were analyzed through a local BLAST (BLASTn and BLASTx, https://blast.ncbi.nlm.nih.gov/Blast.cgi, accessed on 1 March 2025) search to identify viral sequences. Using the online closest astrovirus strain S6.1 (MK404649) as the reference, reference-based mapping and extraction of consensus sequences of astrovirus were performed using CLC Genomics Workbench (Qiagen, Germantown, MD, USA). Sequence alignment was performed using MAFFT version 7 [27], and phylogenetic tree construction was carried out with Molecular Evolutionary Genetics Analysis (MEGA) version 7 [28], while sequence identity calculations were performed using the BioEdit alignment editor (version 7.0.5.3).

## 3. Results

### 3.1. Clinical Signs in Affected Goat Kids

The animal had a history of light yellow, watery diarrhea. Several other goat kids on the same farm had also died exhibiting similar signs. The referring veterinarian reported scant, watery intestinal contents within the small intestine and no other gross lesions.

### 3.2. Histopathology

**Figure 1:**
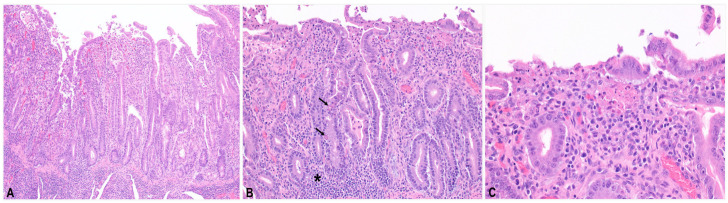
Histopathology of the small intestine. (**A**) The villi are blunted, and crypts are hyperplastic. The epithelium is ulcerated. (**B**) The lamina propria contains many lymphocytes, plasma cells, and macrophages (asterisk). Crypts are irregular. Coccidian intracellular parasite stages (arrows) are present in the crypt epithelium. (**C**) The epithelium is necrotic, with pink pyknotic cells, karyorrhectic debris, and fine cocci.

Microscopically, the small and large intestines exhibited severe necrotizing enterocolitis. In the small intestine (Figure 1), the villi were markedly blunted and crypts were hyperplastic. The epithelium showed multifocal ulcerations with eosinophilic and karyorrhectic debris, pyknotic or karyorrhectic enterocytes and leukocytes, and fine cocci bacteria. Few coccidian intraenterocytic parasites were present in the crypts, ranging from schizonts to zoites. Many crypts were dilated by necrotic debris and eosinophilic mucoid material. The lamina propria contained infiltrates of macrophages, lymphocytes, and plasma cells.

**Figure 2:**
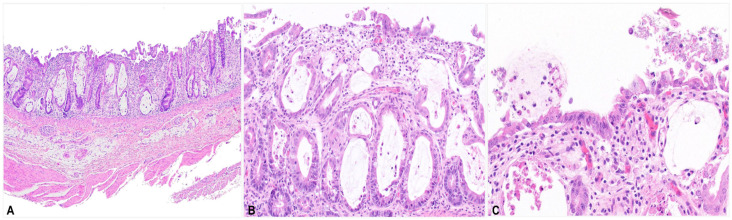
Histopathology of the large intestine. (**A**) The mucosa is irregular with ulceration, crypt dilation, and crypt hyperplasia. The lamina propria is edematous and infiltrated with macrophages, lymphocytes, plasma cells, and fibrosis. (**B**) The crypts are distended with mucus and sloughed cells and are lined by pleomorphic enterocytes. (**C**) The epithelium is eroded, and necrotic cells are evident. Mucus, karyorrhectic leukocytes, enterocytes, and colonies of fine cocci occur along the surface.

In the large intestine (Figure 2), the mucosa was irregular and thin with ulcerations and erosions. Eosinophilic and karyorrhectic debris, mucus, and colonies of fine cocci bacteria occurred along the surface. Crypts were irregular and often dilated by mucus and necrotic enterocytes and lined by either necrotic or hyperplastic epithelium. Many necrotic cells had vacuolated cytoplasm. The lamina propria was fibrotic with macrophages, lymphocytes, and plasma cells scattered throughout. Other tissues lacked significant lesions.

### 3.3. Routine Laboratory Testing Results

Fecal flotation did not reveal organisms. There was heavy growth of *Escherichia coli* and *Enterococcus hirae*. *Salmonella* PCR and rotavirus antigen assays were negative (Table 2).

### 3.4. Metagenomic NGS (mNGS) and Targeted NGS (tNGS) and Genome Analysis

#### 3.4.1. Identification of Goat Astrovirus with Complete Genome

Kraken taxonomical analysis of mNGS data revealed that astrovirus reads (14% of total reads, 111,252 reads in total belonging to *Astroviridae*) were present in the intestinal content (Supplemental Figure S1). The assembled sequence of goat astrovirus IL90175 has two contigs with a 61 bp gap in the capsid gene, as compared to other genomes available on GenBank. In March of 2025, an online NCBI BLAST of the assembled IL90175 astrovirus nucleotide sequence showed that IL90175 had the highest identity (84%) to an ovine astrovirus S6.1 strain from Switzerland with 87% query coverage and 78% identity to a goat strain GS/DX-11/2023 from China (this strain only had capsid gene sequence at GenBank, PQ062281) (Supplementary Figure S2). Further amplicon-based tNGS generated the complete capsid sequence, confirming the capsid sequence derived from mNGS. The complete genome sequence of caprine astrovirus IL90175 strain is 6283 bp in length and deposited into GenBank (accession number: PX492159) and was used for phylogenetic and identity analysis.

#### 3.4.2. Genomic Analysis

In November of 2025, an online NCBI BLAST of the complete genome sequence indicated that caprine astrovirus IL90175 had the highest nucleotide identity (86%) with a newly deposited sheep astrovirus strain, sheep/HA3 (PV400865), with 100% query coverage. This was higher than the identities for the second and third hits: 84% to an ovine astrovirus strain S6.1 (MK404649) and 83% to strain S5.1 (MK404648). Both of these hits showed only 87% query coverage. Whole-genome nucleotide identity analysis using BioEdit revealed that IL90175 shared less than 80% identity with all strains except the highest-identity match, sheep/HA3 (Table 3).

Further analysis of the capsid amino acid sequence using BioEdit showed that caprine astrovirus IL90175 had the highest identity (96.3%) with sheep/HA3, followed by GS/DX-11/2023 (PQ062281) at 86.2%, but markedly lower identities with two ovine strains, S5.1 (62.8%) and S6.1 (60.4%), from Switzerland (Table 3). By contrast, in ORF1a, caprine astrovirus IL90175 showed the highest amino acid sequence identities (94.3%) with both ovine strains S5.1 and S6.1, followed by sheep/HA3 at 91.5%. In ORF1b, IL90175 shared the highest identity with the goat astrovirus G5.1 from Switzerland (96.4%), followed by sheep/HA3 (96.2%) (Table 3).

**Figure 3:**
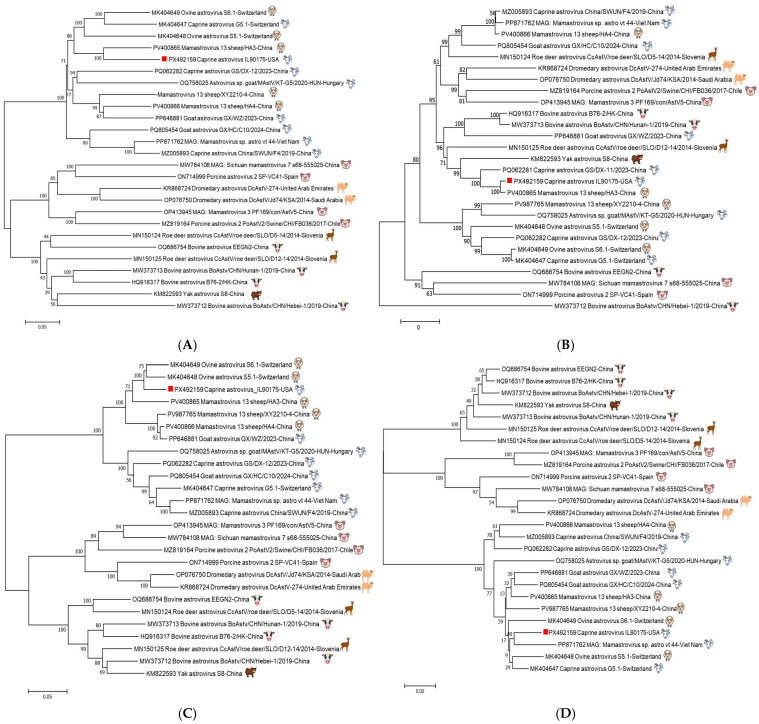
Phylogenetic relationship of caprine astrovirus IL90175 (indicated by the red square) with other representative astroviruses. The country of origin and host species of each virus were provided. The trees were constructed using the neighbor-joining method with the maximum composite likelihood model in MEGA version 17 with 1000 bootstrap replicates, based on the nucleotide sequence of the whole genome (**A**), the amino acid sequence of the capsid protein (**B**), the amino acid sequence of nonstructural polyprotein 1a (**C**), and the amino acid sequence of nonstructural polyprotein 1b (**D**). Icons of animals for all sequences were provided.

Phylogenetic tree analysis showed that IL90175 clustered together with the China sheep/HA3 strain for both complete nucleotide and capsid amino acid sequences (Figure 3A,B). However, IL90175 was more closely related to two sheep strains from Switzerland, S5.1 and S6.1, in the ORF1a amino acid phylogeny, and to the Vietnam goat strain astro_vt_44 in the ORF1b amino acid phylogeny (Figure 3C,D).

## 4. Discussion

Astroviruses are associated with a broad spectrum of diseases: enteric [4,8,9], respiratory [6,19], and neurologic [2,15,16,17,18] across various species. Diarrhea is a major cause of morbidity and mortality in ruminants, particularly in neonates, characterized by dehydration, weight loss, and, in severe cases, death. The description of gastrointestinal disease caused by caprine astrovirus is limited. A study by Wang et al. (China) reported no significant difference in astrovirus detection rates between diarrheic and non-diarrheic samples, and lesions were not described [11]. Additionally, genome characterization of caprine astrovirus is sparse, with only a few strains reported in Switzerland and China in 2019, 2021, 2022, and 2024 [11,20,21,22]. To date, there have been no reports of any genome identification of goat astrovirus in the United States. This study represents the first genome characterization of caprine astrovirus associated with gastrointestinal disease in the U.S.

In this case, co-infection was evident, including coccidia and *E.coli*, which are associated with neonatal diarrhea. The contribution of *E. hirae* to disease is uncertain, as it is uncommonly reported, although a heavy culture growth is seen; this organism is likely an opportunist. The histopathology findings were not typical of bacterial and coccidian infections and suggested viral disease, which spurred metagenomic investigation. It is plausible that the pathogens in this case may play contributory or synergistic roles in disease pathogenesis. Histologic descriptions of astrovirus infection are rarely documented in the literature, especially in ruminants. A study conducted in 1979 by D. R. Snodgrass et al. described histologic lesions in astrovirus-infected lambs, which were limited to mild small intestine lesions and resolved by 5 days post-infection [8]. The villi were atrophied, the crypts were dilated, the enterocytes were lost or necrotic, and the lamina propria was expanded by moderate numbers of macrophages, neutrophils, and lymphocytes [8]. In pigs, the lesions were also found in the small intestine, with villous atrophy, crypt hyperplasia, and neutrophils and macrophages in the villi [29]. Similar histological lesions were observed in our case; however, our findings were more severe, with lesions in the large intestine. Further characterization using in situ hybridization techniques and additional sample examination will better characterize this disease and is being pursued.

New virus/variant discoveries often encounter a significant bottleneck in bioinformatic analysis. The alignment of the initial genome sequence using NCBI online BLAST revealed a large gap in the capsid region of IL90175; however, it mapped to the capsid protein gene of a goat strain, GS/DX-11/2023 (Figure S2). Moreover, mapping raw FASTQ data from mNGS to the closest strain, ovine astrovirus S6.1, using CLC Genomics Workbench revealed a large gap in the capsid region (Figure S3A). To close the sequence gap and verify the capsid sequence, amplicon-based targeted NGS was performed to obtain the complete capsid gene. Using the Qiagen CLC Genomics Workbench, mapping of the raw FASTQ data from mNGS to the complete genome of IL90175 revealed high coverage across the entire genome, including the capsid protein region (Figure S3B). When the closest reference strain sequence was distantly related to the raw FASTQ sequence data, not all astrovirus sequences in the raw reads mapped to the reference (ovine astrovirus S6.1), resulting in gaps in highly divergent regions. These results highlighted the limitations of the online NCBI BLAST program and CLC genomics workbench mapping when analyzing highly divergent sequences.

Recent reports increasingly show that mutational events and recombination events are commonly observed between and within the astrovirus genotypes, especially sheep and goat astroviruses [21,30,31]. In our study, using both NCBI BLAST and BioEdit, the complete genome showed the highest sequence identity to the Mamastrovirus 13 sheep strain recently reported in China in April 2025. Prior to this release, our sequence showed the greatest similarity to the ovine astrovirus S6.1 strain from Switzerland. In the phylogenetic tree, the genome clustered closely with Mamastrovirus 13 sheep and other ovine and caprine strains from Switzerland, China, and Hungary. This finding suggests that the U.S. caprine astrovirus strain shares a common evolutionary origin with astroviruses circulating in Europe and Asia, potentially reflecting cross-species transmission or the global spread of related lineages. In addition to whole-genome comparisons, the capsid amino acid is not only related to caprine astrovirus in China but also closely related to Mamastrovirus 13 in sheep in China, yak astrovirus in China, and roe deer astrovirus in Slovenia. The capsid protein encoded by ORF2 is the most variable region in the genome of mammalian and avian astroviruses, and frequent interspecies transmission and recombination, especially near the ORF1b/ORF2 junction, can disrupt host-specific clustering [32]. The nonstructural genes encoded by ORF1a and ORF1b (including the viral protease and RNA-dependent RNA polymerase) are relatively conserved across related host species [11,30]. The ORF1a/b region in our study supports this concept, clustering with ovine and caprine astroviruses from China, Switzerland, and Vietnam.

In summary, this study reports the first detection of caprine astrovirus in the United States, representing a novel strain of the virus. The affected diarrheic goat exhibited chronic necrotizing enteritis and colitis. However, a significant correlation between astrovirus infection and gastrointestinal disease in goats requires further characterization, as secondary bacterial and protozoal infections were also present in this case. Additional case investigations using RNAscope, broader pathogen screening, continued histopathological analysis, and genome characterization would help clarify the causal relationship between neonatal gastrointestinal disease and astrovirus and reveal the genetic characteristics and diversity of the virus.

## Figures and Tables

**Table 1:** Primer sequence for amplicon-based amplification of the whole capsid.

Primer	Sequence 5′-3′
Cap-Ast-F1	ATGGCTAGTAACAACACACGC
Cap-Ast-R1	ACTATCTGAGCCTGTTGGGT
Cap-Ast-F2	GAGTGGCAGTTTAAGGATTATG
Cap-Ast-R2	CCATGGGAATCTGTGGCCTT
Cap-Ast-F3	CCATCTTGATGAAGGCCACAG
Cap-Ast-R3	TTTCCCCTTCACCTATGCTAAT

**Table 2:** Summary of ancillary testing of the intestinal content sample.

Test	Result
Bacterial culture	Heavy growth of *E. coli* and *E. hirae*
*Salmonella* PCR	Negative
Rotavirus antigen assay	Negative
Fecal flotation	No ova/organism detected

**Table 3:** Amino acid sequence identity (%) in three regions (ORF1a, ORF1b, and capsid), and the complete genome of the caprine astrovirus strain IL90175, compared with other astrovirus strains available online using BioEdit (version 7.0.5.3). Accession numbers and names of reference strains are listed.

Astrovirus Strain	ORF1a	ORF1b	Capsid	Complete Genome ^§^
PV400865_Mamastrovirus_13_sheep/HA3	91.5	96.2	96.3	86.1
PQ062281_Caprine astrovirus isolate GS/DX-11/2023	-	-	86.2	-
KM822593_Yak_astrovirus_S8	67.6	82.6	70.7	65.7
PV400866_Mamastrovirus_13_sheep/HA4	89.4	92.4	69.5	75.7
MN150125_Roe_deer_astrovirus_CcAstV/roe_deer/SLO/D12-14/2014	68.6	83.2	68.2	66.7
MZ005893_Caprine_astrovirus_China/SWUN/F4/2019	84.3	94.2	68.2	73.9
PQ805454_Goat_astrovirus_GX/HC/C10/2024	85.1	95.8	68.2	75.0
PP871762_MAG:_Mamastrovirus_sp._astro_vt_44	84.7	95.6	67.5	74.7
HQ916317_Bovine_astrovirus_B76-2/HK	67.1	84.2	65.2	65.8
MW373713_Bovine_astrovirus_BoAstv/CHN/Hunan-1/2019	66.1	84.2	64.9	66.2
MK404648_Ovine_astrovirus_S5.1	**94.3**	95.8	62.8	75.7
MN150124_Roe_deer_astrovirus_CcAstV/roe_deer/SLO/D5-14/2014	67.2	84.0	62.3	64.7
MK404647_Caprine_astrovirus_G5.1	84.7	**96.4**	60.7	73.0
MK404649_Ovine_astrovirus_S6.1	**94.3**	95.8	60.4	76.7
PQ062282_Caprine_astrovirus_GS/DX-12/2023	85.3	94.4	60.3	73.1
PV987765_Mamastrovirus_13_sheep/XY2210-4	88.9	95.6	59.8	72.9
PP646881_Goat_astrovirus_GX/WZ/2023	88.6	95.6	59.4	75.5
KR868724_Dromedary_astrovirus_DcAstV-274	63.8	81.4	55.5	62.9
OQ758025_Astrovirus_sp._goat/MAstV/KT-G5/2020-HUN	82.4	92.4	54.6	70.4
MZ819164_Porcine_astrovirus_2_PoAstV2/Swine/CHI/FB036/2017	65.8	80.6	54.5	62.4
OP076750_Dromedary_astrovirus_DcAstV/Jd74/KSA/2014	63.9	80.6	54.1	62.3
OP413945_MAG:_Mamastrovirus_3_PF169/con/AstV5	64.7	80.4	53.8	62.7
OQ686754_Bovine_astrovirus_EEGN2	68.2	83.6	48.0	62.9
ON714999_Porcine_astrovirus_2_SP-VC41	62.7	81.4	47.5	61.5
MW784108_MAG:_Sichuan_mamastrovirus_7_s68-555025	65.2	81.6	44.3	60.6
MW373712_Bovine_astrovirus_BoAstv/CHN/Hebei-1/2019	68.2	84.0	42.1	59.8

^§^: nucleotide sequences of complete genome; -: data is not applicable since this caprine astrovirus isolate GS/DX-11/2023 (PQ062281) only has the capsid gene available; MAG: metagenome-assembled genome. The strains with the highest amino acid identities with IL90175 in ORF1a and ORF1b had their values bolded.

## Data Availability

Related sequence data were deposited into GenBank, accession number: PX492159.

## Supplementary Materials

The following supporting information can be downloaded at https://www.mdpi.com/article/10.3390/v18010120/s1, Figure S1. Kraken taxonomical classification of raw FASTQ data generated from metagenomic sequence revealed the presence of astroviruses in the sample. Figure S2: Online BLAST result of the whole genome before Mamastrovirus 13 sheep/HA3 (PV400865) became available. Figure S3. Using Qiagen CLC Workbench, mapping all FASTQ raw read sequences to an online reference strain of ovine astrovirus S6.1 (A) and the caprine astrovirus IL90175 (B).
