# Supplementary material for: Histopathologic and Genomic Characterization of a Novel Caprine Astrovirus Identified in a Boer Goat Kid in Illinois, United States
Source: Viruses. 2026 Jan 16;18(1):120. doi: 10.3390/v18010120 (PMC12846467; doi:10.3390/v18010120)
Supplement: Supplementary file 1 [file viruses-18-00120-s001.zip › viruses-4037660-supplementary.pdf]

Supplementary Materials:

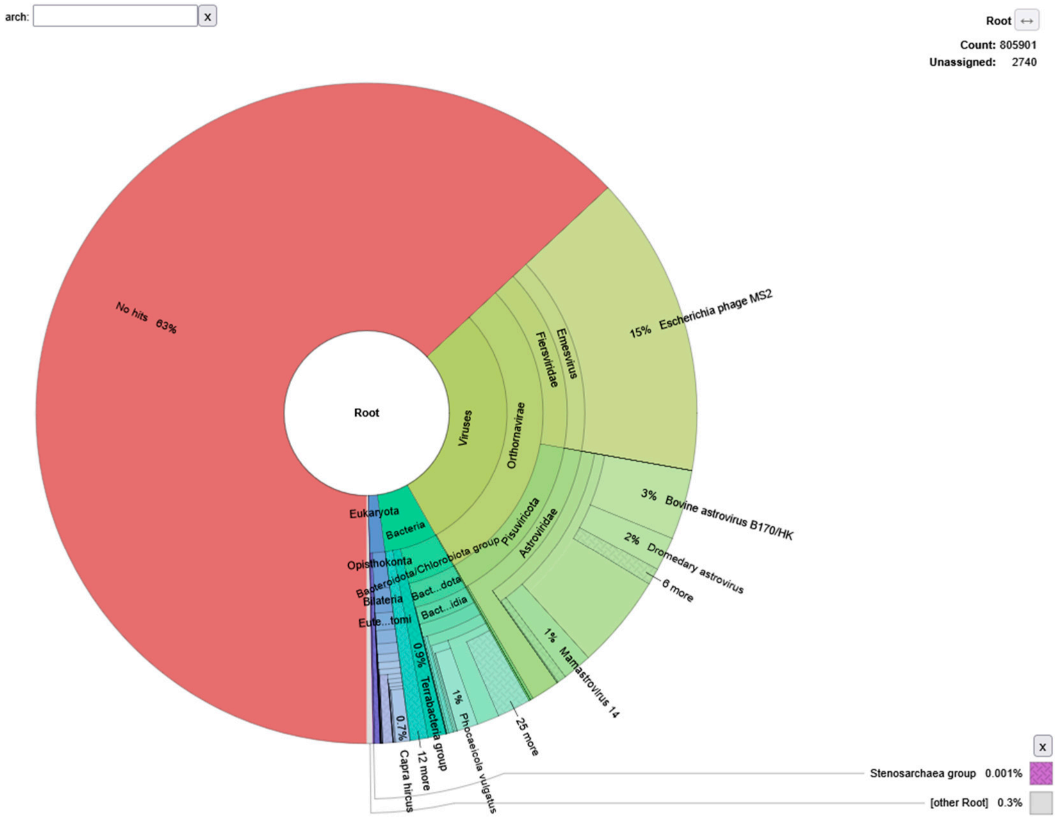

Figure S1. Kraken taxonomical classification of raw FASTQ data generated from metagenomic sequence revealed the presence of astroviruses in the sample.

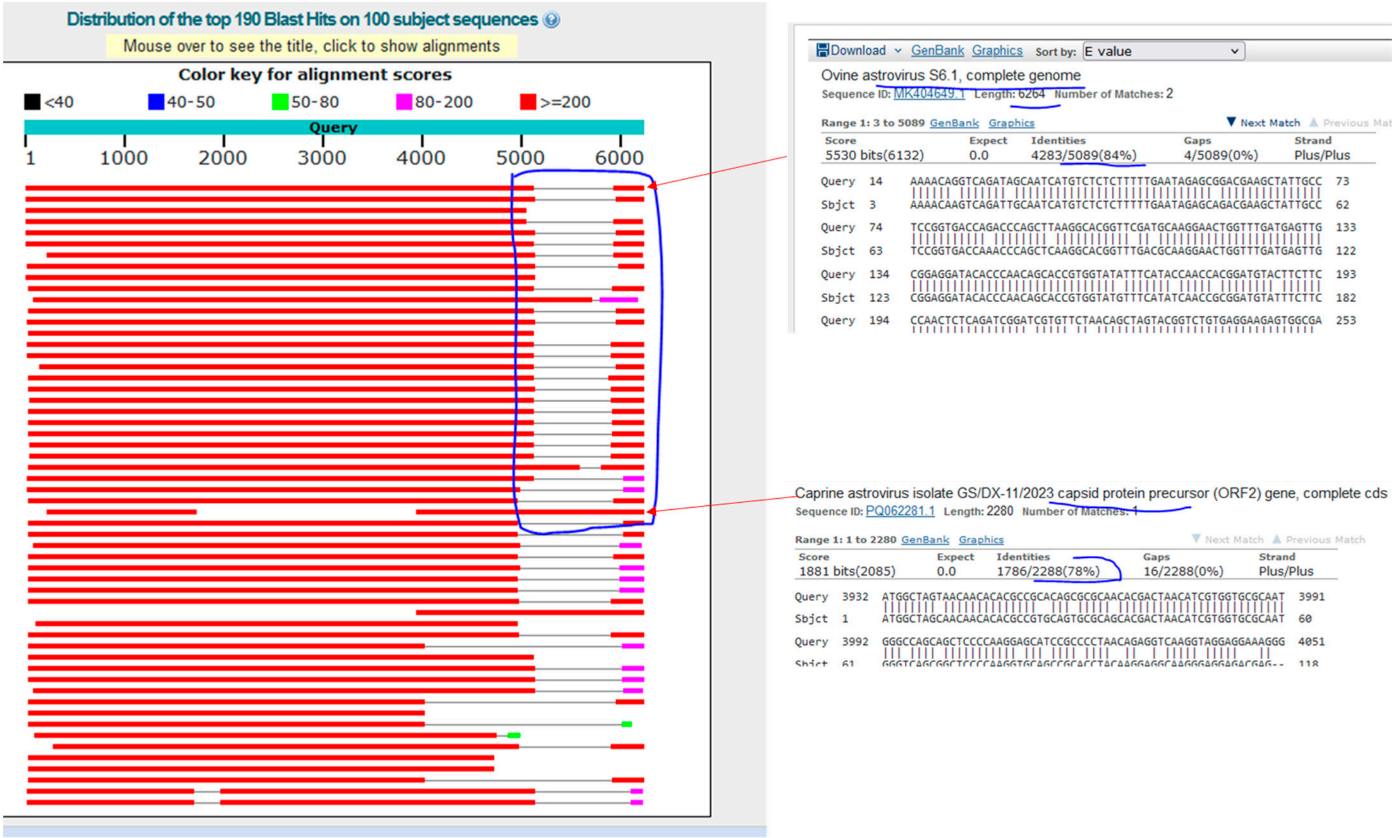

Figure S2: Online BLAST result of the whole genome before Mamastrovirus 13 sheep/HA3 (PV400865) became available. The gap was shown as a grey line highlighted within the blue box, suggesting regions that did not align between our query and subject sequences. However, the capsid region aligned with caprine astrovirus capsid protein ORF2 gene of GS/DX-11/2023 strain (PQ062281). The additional alignment within the first 2,000 nt of the GS/DX-11/2023 sequence was actually from a different strain, astrovirus-2023-Gansu (PQ573797), due to an incorrect alignment.

(A)

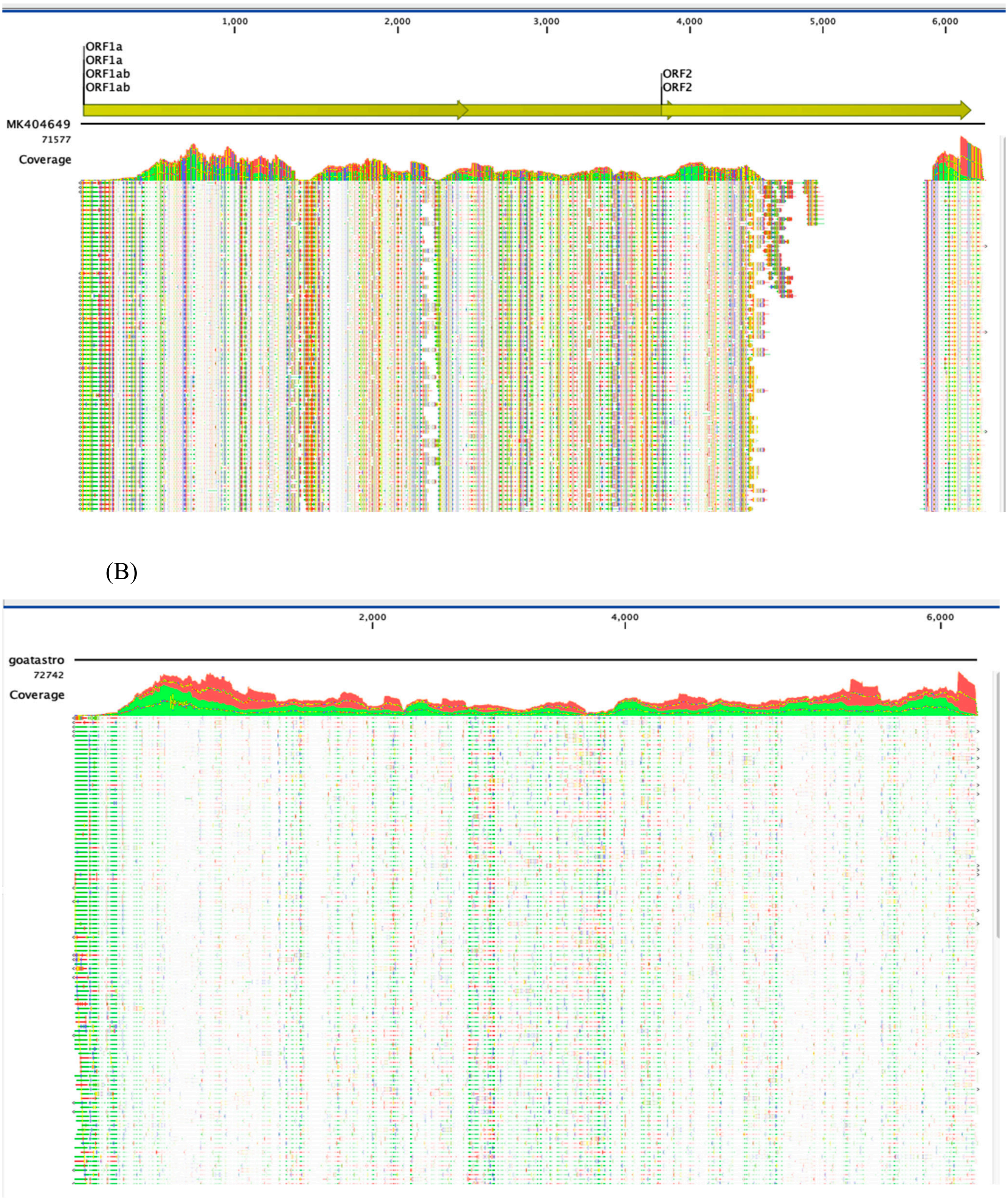

(B)

Figure S3. Using Qiagen CLC Workbench with default settings, mapping all FASTQ raw read sequences to an online reference strain of ovine astrovirus S6.1 (A) and the caprine astrovirus IL90175 (B). The capsid region was not mapped when compared with Ovine astrovirus S6.1 but was successfully identified when aligned with the complete genome IL90175 with its capsid gene sequence verified through amplicon-based sequencing.
